# Prion degradation pathways: Potential for therapeutic intervention

**DOI:** 10.1016/j.mcn.2014.12.009

**Published:** 2015-05

**Authors:** Rob Goold, Chris McKinnon, Sarah J. Tabrizi

**Affiliations:** a Department of Neurodegenerative Disease, UCL Institute of Neurology, University College London, United Kingdom

**Keywords:** Prion disease, PrP^Sc^, Autophagy, Proteasome, Lysosomal degradation, Therapeutics

## Abstract

Prion diseases are fatal neurodegenerative disorders. Pathology is closely linked to the misfolding of native cellular PrP^C^ into the disease-associated form PrP^Sc^ that accumulates in the brain as disease progresses. Although treatments have yet to be developed, strategies aimed at stimulating the degradation of PrP^Sc^ have shown efficacy in experimental models of prion disease. Here, we describe the cellular pathways that mediate PrP^Sc^ degradation and review possible targets for therapeutic intervention. This article is part of a Special Issue entitled ‘Neuronal Protein’.

## 1 Introduction

Prion diseases are thought to be caused by the misfolding of native cellular prion protein (PrP^C^) into a β-sheet rich aggregation prone form (PrP^Sc^). Their pathogenesis is associated with the build-up of PrP^Sc^ in the brains of affected individuals (Prusiner, 1998). As a result, prion diseases are included in a group of neurodegenerative disorders termed the proteinopathies, alongside Alzheimer's disease (AD), Parkinson's disease (PD) and Huntington's disease (HD) (Soto, 2003). The abnormal protein aggregates which accumulate in these disorders are thought to result in a toxic gain of function that ultimately leads to cell death and disease pathogenesis. Debate about the nature of these toxic effects is ongoing (Lindquist and Kelly, 2011); however, recent evidence has emerged implicating impaired protein homeostasis (proteostasis) as a major cause of toxicity common to these disorders (Hetz and Mollereau, 2014; Lindquist and Kelly, 2011). To function efficiently, cells must maintain protein content (proteome) in an active state. This presents a significant challenge given the inherently unstable nature of many proteins under physiological conditions. Proteostasis is defined as the balance between the protein degradation and synthesis needed to remove and replace denatured proteins, respectively. Almost 1400 proteins (~ 14% of the proteome) regulate proteostasis in mammalian cells, as part of a tightly co-ordinated proteostasis network (Kim et al., 2013; Powers et al., 2009).

Protein translation is regulated by a series of initiation and elongation factors. One of the key regulators is eIF2α (Walter and Ron, 2011) which is targeted by a number of signal transduction pathways known to control protein synthesis (Clemens, 2004; Deng et al., 2002; Harding et al., 1999). Phosphorylation of eIF2α inhibits its activity and suppresses global protein synthesis (Walter and Ron, 2011). This pathway forms a key arm of the unfolded protein response (UPR), which is activated during conditions of cellular stress. The UPR has been shown to be particularly significant in prion pathology (Hetz and Mollereau, 2014; Moreno et al., 2012).

Once translated, proteins are scrutinised for correct folding by multiple quality control pathways. In the cytosol, the hsp70/hsp40 chaperone system (Kim et al., 2013) surveys proteins for exposed hydrophobic regions found in misfolded proteins. If attempts at refolding fail, misfolded proteins are targeted for degradation. For secretory or membrane proteins which are translocated directly into the endoplasmic reticulum (ER) during synthesis (cotranslational translocation), specialised quality control systems operate within the ER lumen (ERQC). Here, the situation is more complex than in the cytosol due to the additional need to monitor signal peptide removal, N-linked glycosylation, and disulphide bond formation (Braakman and Hebert, 2013). Since the ER lumen lacks degradation machinery, misfolded proteins must be retro-translocated to the cytosol for degradation as part of the ER-associated degradation (ERAD) pathway. Irreversibly aggregated ER proteins are subject to ERQC and targeted for lysosomal degradation via autophagic pathways (Araki and Nagata, 2011). In addition to ERAD and ERQC pathways, it is likely that protein quality control systems in other cellular compartments also contribute to the clearance of misfolded proteins. An important example is the Golgi quality control (Golgi QC) pathway which directs misfolded proteins from the Golgi directly to lysosomes for degradation (Anelli and Sitia, 2008; Arvan et al., 2002).

Misfolded, damaged or aggregated mature proteins are subject to similar quality control mechanisms as those synthesised de novo (Hipp et al., 2014). Protein aggregates accumulate in cells when levels of misfolded proteins overwhelm the quality control systems. This can arise in conditions of cell stress, mutant protein expression or prion infection. Different classes of protein inclusions have been described depending on their cellular location, stability and protein content. They are thought to play a protective role by sequestering potentially harmful misfolded proteins from the cellular milieu (Sontag et al., 2014). Various systems have evolved to deal with these deposits. Hsp70, Hsp40 and Hsp100 chaperones act in concert to solubilise aggregates, allowing refolding or degradation (Kim et al., 2013). Insoluble aggregates are directly targeted for degradation by binding to adaptor proteins, such as p62 and NBR1 (Bjorkoy et al., 2005; Kirkin et al., 2009; Pankiv et al., 2007). The eventual fate of terminally misfolded or aggregated proteins is degradation. There are two main degradation pathways: the ubiquitin-proteasome system (UPS) and lysosomal proteolysis (including autophagic pathways). These systems are particularly important in neurons whose complex architecture, long lifespan and inability to divide (and thereby dilute the load of damaged proteins), make them particularly vulnerable to proteotoxic stress.

## 2 Ubiquitin–proteasome system

As the principal route of protein degradation in mammalian cells, the UPS represents a major protection against misfolded proteins. Proteins are marked for proteasomal degradation by covalent conjugation of ubiquitin (Ub) in a sequential reaction involving three enzymes: ubiquitin activating enzymes (E1), ubiquitin conjugating enzymes (E2) and ubiquitin ligases (E3) that recognise and transfer ubiquitin to an internal lysine residue on substrate proteins. In humans, there are two E1 molecules, a greater diversity of E2s, and several hundred E3s (Lee et al., 2011). Thus, E3 ubiquitin ligases provide the mechanisms of substrate specificity in proteasomal degradation. Following initial substrate ubiquitination further Ub molecules are added sequentially to the first via one of seven internal lysine residues. In addition to canonical lysine 48 linkages, lysine 11 and 29 linkages have been shown to target proteins for proteasomal degradation, with a chain of four molecules considered the minimum efficient signal (degron) for recognition by the 26S proteasome (Dantuma and Bott, 2014; McKinnon and Tabrizi, 2014). This large (2.5 MDa) multi-subunit complex consists of a barrel-shaped 20S catalytic core responsible for proteolytic activity (Groll et al., 2000) and the 19S regulatory particle, which is important for the recognition, unfolding, and translocation of ubiquitinated substrates into the 20S core particle (Bedford et al., 2010). Mutations in different components of the UPS have been identified in clinical cases of HD, AD and PD (Kitada et al., 1998; van Leeuwen et al., 2006). Furthermore, experimental knockout of proteasome subunits in mice has been shown to result in progressive neurodegeneration, clearly demonstrating the importance of proteasome catalytic activity to neuronal proteostasis and survival (Bedford et al., 2008; Tashiro et al., 2012). Ageing has also been linked with a reduction in UPS activity, a factor that may contribute to the late onset of many neurodegenerative diseases (Gamerdinger et al., 2009; Tydlacka et al., 2008; Zhou et al., 2003).

Although implicated in the clearance of many disease-associated proteins (Bhat et al., 2014; Goold et al., 2013; Li et al., 2010), proteasomal degradation may be restricted to soluble misfolded proteins or smaller oligomeric forms that can be unfolded to allow entry into the 20S catalytic chamber. For larger, more insoluble aggregates, the catalytic chamber may remain inaccessible, preventing their effective degradation (Qin et al., 2003; Scotter et al., 2014). Indeed, many oligomeric and aggregated forms of disease-associated proteins have been shown to inhibit proteasome activity, both in reconstituted systems using purified components, as well as in cultured cells and in vivo models (Andre and Tabrizi, 2012; Deriziotis et al., 2011; Hong et al., 2014; Kristiansen et al., 2007). In the context of UPS impairment, an upregulation of autophagy has been described, which may facilitate the clearance of larger aggregates (Korolchuk et al., 2010). This is a good example of the cross-talk and close interplay thought to exist between the two degradatory systems (Hao et al., 2013; Nedelsky et al., 2008).

## 3 Lysosomal degradation/autophagy

Lysosomes represent the major catabolic compartment in eukaryotic cells. A wide range of enzymatic activities are confined within the lysosomal limiting membrane. These include many classes of proteolytic enzymes (Appelqvist et al., 2013). Several routes deliver cell constituents to lysosomes including endolysosomal pathways mediated by the ESCORT machinery, as well as ERQC and Golgi QC pathways and autophagic pathways (Saftig and Klumperman, 2009). These systems are interlinked and crosstalk between them ensures the efficient removal of obsolete cellular components (Nixon, 2013).

Autophagy is a highly conserved system for the degradation of cytosolic macromolecules and organelles. Several pathways have been described with the most important for neuronal proteostasis being macroautophagy (Jimenez-Sanchez et al., 2012; Nixon, 2013; Yao et al., 2013). This is a process whereby cytosolic contents are engulfed in a double membrane-bound structure, called an autophagosome, which later fuses with lysosomes to enable degradation to take place. The process begins with formation of a crescent shaped isolation membrane (phagophore). The isolation membrane then extends around a region of cytoplasm or selected substrate. Subsequent closure of the inner and outer bilayers of the isolation membrane forms the autophagosome, which later fuses with a lysosome to yield an autolysosome (Rubinsztein et al., 2012). The mammalian target for rapamycin complex (mTORC) is an important negative regulator of autophagy whose activity is influenced by multiple signalling pathways (Rubinsztein et al., 2012). However, mTORC-independent pathways have also been described that involve Beclin 1 and the PI3K vps34 (Sarkar et al., 2005; Williams et al., 2008).

The importance of autophagy to neuronal proteostasis was shown by a mouse conditional knockout of atg5, a key autophagy intermediate, in the CNS. On atg5 deletion, mice developed behavioural deficits and neurodegeneration (Hara et al., 2006). Interestingly, affected mice also accumulated abnormal ubiquitinated proteins which led to the formation of aggregates in neurons (Hara et al., 2006). Induction of autophagy has been shown to be beneficial in many models of neurodegenerative disease through the degradation of aggregation-prone mutant proteins including Huntingtin (Ravikumar et al., 2004), *α*-Synuclein (Webb et al., 2003), APP (Spilman et al., 2010), Tau (Ozcelik et al., 2013) and TDP-43 (Wang et al., 2012).

## 4 Prion disease and proteostasis

To date, many studies have identified evidence of proteostasis dysregulation in prion disease. Early reports demonstrated the presence of abnormal levels of ubiquitin and ubiquitinated proteins in diseased mouse brain tissue (Kenward et al., 1994; Lowe et al., 1992). More recent studies have confirmed the presence of ubiquitin-positive staining in the form of intracellular inclusions or prominent extracellular puncta in the brains of diseased animals (Kristiansen et al., 2007). The abnormal levels of ubiquitinated protein indicate a failure of protein degradation pathways. Accumulation of proteasomal substrates at later stages of disease correlate with a decrease in proteasome catalytic activity in brain extracts from diseased animals (Deriziotis et al., 2011; Kristiansen et al., 2007). Transgenic mice expressing the UPS reporter Ub^G76V^-GFP showed strong reporter accumulation in the brain regions worst affected by prion disease, supporting a role for proteasomal impairment in disease pathogenesis (Kristiansen et al., 2007).

Abnormalities in the lysosomal system have also been observed in prion diseases. Increases in the number and size of autophagic vacuoles were reported in the brains of patients affected by prion disease, as well as in mouse models, suggesting that autophagy may be up-regulated in prion disease (Boellaard et al., 1991; Liberski et al., 2010; Sikorska et al., 2004). Consistent with this hypothesis, an increase in p62 expression in diseased brain was recently reported and may reflect attempts to increase the clearance of aggregated proteins by autophagy (Homma et al., 2014).

In addition to impairments in degradation systems, many studies have indicated that ER stress is a feature of prion disease in both human patients and animal models (Hetz et al., 2003), with many UPR markers upregulated relatively early in disease pathogenesis (Hetz and Soto, 2006; Moreno et al., 2012; Rane et al., 2008). Moreover, mechanistic studies have shown that prion infection induces a global down-regulation of protein translation through chronic eIF2α phosphorylation (Moreno et al., 2012) and ER protein translocation impairment (Rane et al., 2008). Thus, signs of ER stress appear pre-symptomatically and have been suggested as important mediators of prion toxicity (Hetz and Soto, 2006). However, the causal relationship between these observations and disease pathogenesis is currently unknown. Misfolded PrP in the ER could induce ER stress (Hetz and Mollereau, 2014). Alternatively, the accumulation of PrP in aggresomes may sequester cytosolic components leading to proteostatic impairment (Chakrabarti and Hegde, 2009; Kristiansen et al., 2007). Complicating the picture further is the close relationship that exists between the UPS, autophagy and ER function (Dantuma and Bott, 2014; Hetz and Mollereau, 2014). For example, there is a reciprocal relationship between ER stress and proteasome activity, such that proteasome inhibition has been shown to induce ER stress and vice versa (Lee et al., 2003; Menendez-Benito et al., 2005). Hence, deciphering which, if any of these factors, is causal to disease pathogenesis presents a significant challenge. Despite this, it is clear that disease pathogenesis is intimately linked to ongoing PrP^Sc^ propagation (Aguzzi and Falsig, 2012) and that lowering PrP^Sc^ load increases the lifespan of prion-infected mice (Mallucci et al., 2003; Mallucci et al., 2007). As a result, prion degradation pathways may represent a viable therapeutic target for the treatment of prion diseases.

## 5 Prion degradation pathways

**Fig. 1:**
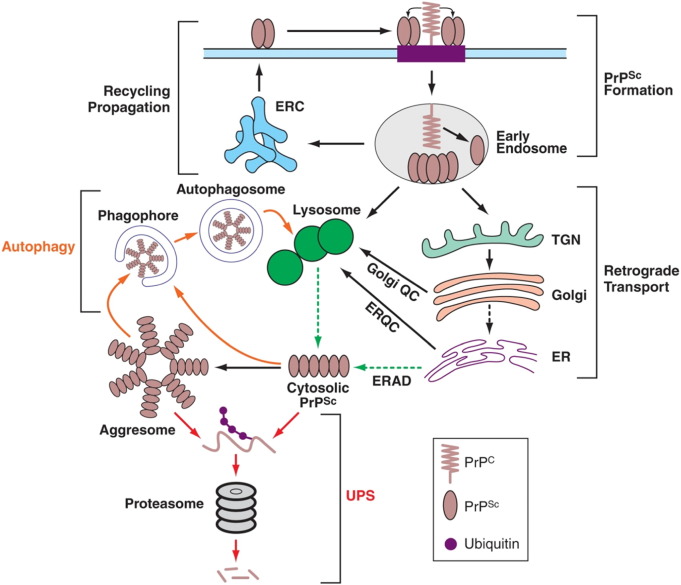
PrP^Sc^ formation, trafficking and degradation. Schematic illustrating PrP^Sc^ metabolism. PrP^Sc^ forms at the plasma membrane or shortly after endocytosis in endosomes, the ERC or lysosomes. Recycling of PrP^Sc^ to the plasma membrane allows prion propagation. Newly formed PrP^Sc^ undergoes retrograde transport to the trans Golgi network (TGN) and Golgi where it is subject to Golgi quality control and trafficked to lysosomes for degradation. More mature forms of PrP^Sc^ are trafficked to lysosomes via the endolysosomal and autophagic pathways. PrP^Sc^ may reach the cytosol through lysosomal rupture or ERAD, and accumulates in aggresomes under conditions of proteasome impairment. Unfolding and ubiquitination precede proteasomal degradation (UPS pathways shown in red). Aggresomal PrP^Sc^ and smaller insoluble forms are engulfed by phagophores and degraded by autophagic pathways (shown in orange).

In vivo observations support a role for both the lysosomal system and the UPS in prion degradation. Several studies have reported that the majority of intracellular PrP^Sc^ is found in the endolysosomal system (Jeffrey et al., 2010 and therein). In prion-infected brain tissue, increased numbers and sizes of late endosomes, lysosomes and autophagic vesicles have been described (Boellaard et al., 1991; Liberski et al., 2010; Sikorska et al., 2004). Dual-labelling experiments also confirmed the colocalisation of PrP^Sc^ with lysosomal markers (DeArmond and Bajsarowicz, 2010). Interestingly, reports of N-terminal truncation of PrP suggest that lysosomes play an active role in PrP^Sc^ degradation (Jeffrey et al., 2003). In addition to the lysosomal system, we have previously reported a biochemical association between PrP^Sc^, 20S proteasome subunits and other cytosolic aggresome markers (Hsp70 and vimentin) in prion-infected mouse brain (Kristiansen et al., 2005). This is of particular interest since aggresomes are thought to sequester misfolded proteins and target them for degradation by both the UPS and autophagy (Dantuma and Bott, 2014; Sontag et al., 2014). Thus, the two major protein clearance pathways appear to be involved in prion degradation (Fig. 1).

These in vivo findings were largely confirmed by in vitro experiments in various neuroblastoma and other cultured cell lines which stably propagate prions. The potential for genetic and pharmacological manipulation of cultured cells has facilitated a more detailed analysis of PrP^Sc^ intracellular trafficking and degradation pathways. PrP^Sc^ is found on the plasma membrane, in the endolysosomal system, the endosomal recycling compartment, the trans Golgi network and Golgi (via retromer mediated retrograde transport), in the autophagic pathway and in the cytosol (Beranger et al., 2002; Borchelt et al., 1992; Magalhaes et al., 2005; Marijanovic et al., 2009; Rouvinski et al., 2014; Veith et al., 2009; Yamasaki et al., 2014). Much of the work on PrP^Sc^ intracellular distribution was directed at finding the site of prion conversion (i.e., the templated misfolding of native PrP^C^ into PrP^Sc^). Despite useful information provided by these studies, they rarely examined prion degradation directly. This is important because the PrP^Sc^ content of a cell at any instant reflects the fluctuating balance between synthesis (i.e., new prion conversion) and degradation. The wide variety of compounds known to down-regulate PrP^Sc^ levels in cultured cells with no apparent commonality in their mode of action gives an indication of the complexity of prion metabolism (Trevitt and Collinge, 2006). Hence, the overall PrP^Sc^ content of a cell is not solely a reflection of its degradation rate and should not be interpreted as such. The situation is further complicated by the observation that treatments which block PrP^Sc^ degradation often lead to an increase in PrP^C^ levels (Nunziante et al., 2011). Higher cellular levels of PrP^C^ are likely to promote prion conversion and increase PrP^Sc^ levels independent of any block in its degradation (Nishida et al., 2000). The converse is also likely to be true, whereby agents which reduce levels of PrP^Sc^ also deplete PrP^C^ (Goold et al., 2013; Heiseke et al., 2009). It is therefore necessary to interpret data regarding total PrP^Sc^ levels with caution when considering possible mechanisms of degradation.

Recent work looking directly at the degradation of surface-labelled PrP^Sc^ has demonstrated an important role of the lysosome in prion degradation (Goold et al., 2013). Autophagy appears to be the major route of PrP^Sc^ delivery to lysosomes, at least in chronically-infected cells (Heiseke et al., 2010; Yao et al., 2013). Genetic ablation of key autophagic components and pharmacological blockade both increase PrP^Sc^ levels (Goold et al., 2013; Heiseke et al., 2009; Heiseke et al., 2010). Conversely, stimulating autophagy has been shown to decrease PrP^Sc^ load (Aguib et al., 2009; Heiseke et al., 2010; Homma et al., 2014). Other non-autophagy dependent routes of lysosomal delivery have also been proposed. Yamasaki and colleagues suggested that upon prion exposure, N2a cells channel a significant proportion of newly endocytosed PrP^Sc^ through the endolysosomal pathway for rapid degradation (Yamasaki et al., 2014). Similar findings have previously been reported in primary dorsal root ganglion neurons (Jen et al., 2010). The Golgi QC pathway has been shown to be important for the clearance of some PrP genetic mutants and newly synthesised PrP^Sc^ (Ashok and Hegde, 2009; Goold et al., 2013). Taken together, the complexity of prion degradation likely reflects differences in the cell types used and forms of misfolded PrP being studied (e.g., mutant PrP isoforms, newly-formed PrP^Sc^ and mature PrP^Sc^).

In addition to lysosomal and autophagic degradation pathways, our recent work suggests that the UPS also plays an important role in PrP^Sc^ degradation. In chronically-infected cultured cells, we found that application of proteasome inhibitors precipitated a rapid rise in PrP^Sc^ levels, with detectable increases as early as three hours post-application (Goold et al., 2013). Importantly, elevated PrP^Sc^ levels were not accompanied by increased PrP^C^ expression, suggesting that PrP^Sc^ degradation itself was the treatment target. Interestingly, proteasomal inhibition has been shown to lead to aggresome formation in many cell types (Kawaguchi et al., 2003). In prion-infected cells these perinuclear inclusions contain PrP^Sc^ and other typical aggresome markers including Hsp70, proteasome subunits and vimentin, (Kristiansen et al., 2005). These pharmacologically-induced aggresomes suggest the presence of cytosolic PrP^Sc^ in cultured cells (Ben Gedalya et al., 2011; Dron et al., 2009; Kristiansen et al., 2005). This is an important observation since proteasomal activity is restricted to the cytosol and nucleus (McKinnon and Tabrizi, 2014) and PrP^Sc^ must therefore access one of these compartments to be considered a direct proteasomal substrate.

As an outer leaflet membrane protein, mature PrP would not normally gain access the cytosol. Since prion conversion occurs after PrP maturation (Borchelt et al., 1990; Caughey and Raymond, 1991) at the plasma membrane (Goold et al., 2011), and/or following endocytosis (Beranger et al., 2002; Borchelt et al., 1992; Caughey et al., 1991; Marijanovic et al., 2009; Yamasaki et al., 2014), PrP^Sc^ must traverse the plasma membrane or an intracellular membrane to gain access to the cytosol. How and where this process takes place remains unclear, yet various mechanisms can be envisaged. The build-up of aggregated PrP^Sc^ in lysosomes may de-stabilise the membrane, causing membrane leakage of the lumen contents into the cytosol, an event which has previously been described for other disease related proteins (e.g. Micsenyi et al., 2013). Alternatively, PrP^Sc^ may act as an ERAD substrate, as has been described for certain PrP mutant forms (Jin et al., 2000; Zanusso et al., 1999).

Once in the cytosol, PrP^Sc^ ubiquitination and unfolding are likely prerequisites for proteasomal degradation. Although ubiquitin-independent pathways to proteasomal degradation have been described (Finley, 2009), most substrates require ubiquitination for efficient recognition (Bhattacharyya et al., 2014). Evidence that PrP can be ubiquitinated has been hard to come by. In vivo, highly sensitive methods were required to detect ubiquitinated PrP, which was restricted to larger PrP^Sc^ aggregates present at late stages of disease (Kang et al., 2004; Kovacs et al., 2005). Although ubiquitin antibodies stain PrP-enriched aggresomes that form following proteasome inhibition in prion-infected cells (Kristiansen et al., 2005), only a low level of colocalisation between PrP^Sc^ and ubiquitin immunostaining in vivo have been reported (Cammarata and Tabaton, 1992). Hence, PrP^Sc^ does not seem to be ubiquitinated to a significant degree and its status as a genuine proteasome substrate remains open to debate. It is possible that most PrP^Sc^ remains non-ubiquitinated and becomes sequestered in Q-bodies — small, dynamic protein quality control compartments shown to form under basal conditions in cultured cells (Escusa-Toret et al., 2013; Sontag et al., 2014). Q-bodies may coalesce to form aggresomes under conditions of greater cell stress such as those prevalent during pharmacological proteasome inhibition or in the later stages of prion disease (Grenier et al., 2006; Kristiansen et al., 2005). Unfolding and ubiquitination of the Q-body PrP^Sc^ population could instigate rapid degradation. Ubiquitinated PrP^Sc^ would thus represent only a small proportion of total PrP^Sc^ at steady state and could remain below detection thresholds. In addition to direct degradation, the proteasome may also regulate PrP^Sc^ levels indirectly through clearance of PrP^C^ thereby reducing the substrate levels for prion synthesis (Yedidia et al., 2001). Decreasing the rate of PrP^Sc^ synthesis may allow alternative degradative systems to reduce the levels of pre-existing PrP^Sc^ independent of, or in conjunction with, direct UPS activity.

Recent studies have highlighted the dynamic nature of PrP^Sc^ metabolism. Significant increases in PrP^Sc^ levels can be induced by a blockade of degradative activity which lasts only a few hours (Goold et al., 2013). Similarly, stimulation of these pathways clears prions from cells rapidly (Ertmer et al., 2004). Early metabolic labelling experiments suggested that much of the total cellular PrP^Sc^ content is relatively stable (Boellaard et al., 1991; Caughey and Raymond, 1991). However, surface-labelling experiments revealed that PrP^Sc^ on the plasma membrane is highly labile (Caughey and Raymond, 1991; Goold et al., 2013). This suggests that there are two populations of PrP^Sc^ within the infected cell: a plasma membrane population (including newly formed PrP^Sc^) which is metabolised rapidly, and a more stable, and possibly more aggregated, internalised population which comprises the majority of total cellular PrP^Sc^. We found that newly formed PrP^Sc^ is a substrate for non-autophagy dependent lysosomal degradation (i.e., the Golgi QC pathway) (Goold et al., 2013). In contrast, PrP^Sc^ from chronically-infected cells is also subject to UPS and autophagy-dependent lysosomal degradation (Goold et al., 2013; Heiseke et al., 2009; Heiseke et al., 2010; Yao et al., 2013). This difference in metabolic fates may be due to differential trafficking of PrP^Sc^ in cells with established prion propagation (Yamasaki et al., 2014). Alternatively, it could be explained by maturation of PrP^Sc^ into a more aggregated state or its de novo appearance in the cytosolic compartment which, as previously discussed, is a necessary prerequisite for UPS-mediated degradation.

Significantly, induction of autophagy has been shown to reduce total cellular PrP^Sc^ levels rapidly (Ertmer et al., 2004). This indicates that stimulation of cellular degradation systems is sufficient to overcome the apparent stability of PrP^Sc^ levels under steady state conditions (Ertmer et al., 2004; Goold et al., 2013). It is also interesting to note that some treatments which have been shown to reduce PrP^Sc^ load in cultured cells were also shown to be effective in vivo, both in terms of a reduction in PrP^Sc^ load and clinical outcome (Yao et al., 2013).

## 6 Therapeutics

**Fig. 2:**
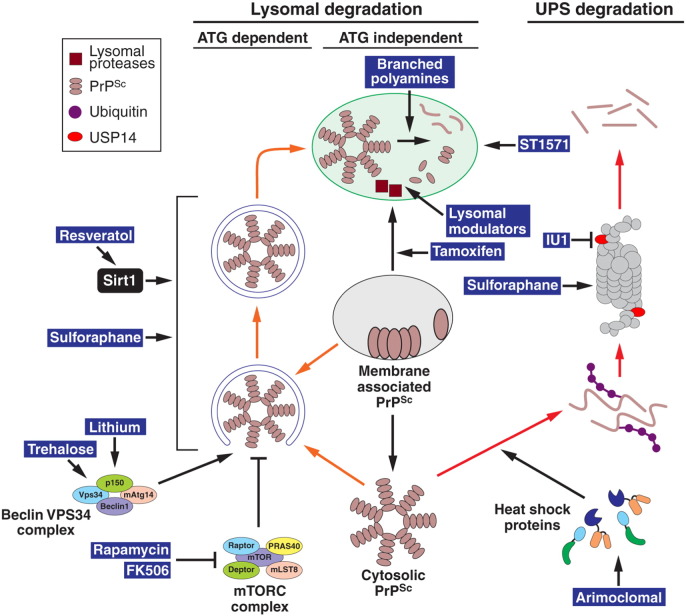
Therapeutic targets in PrP^Sc^ degradation pathways. Membrane associated PrP^Sc^ is trafficked to lysosomes for degradation through endolysosomal, Golgi quality control or autophagic pathways. Cytosolic PrP^Sc^ degradation is mediated by autophagy (orange arrows) and the UPS (red arrows). Reagents known to enhance the activity of these pathways are shown in blue highlights. Identified target proteins are indicated (details in the text).

Prion diseases are fatal neurodegenerative disorders that include Creutzfeldt–Jakob disease (CJD), Gerstmann–Straussler–Scheinker syndrome, kuru and fatal familial insomnia. To date, no therapeutic or prophylactic regimens exist for these disorders. A variety of therapeutic strategies have been proposed, with most directed at preventing prion conversion. One approach is to reduce PrP^C^ expression or trafficking to the plasma membrane, reducing its availability for prion conversion (Gilch et al., 2001; Tilly et al., 2003). Alternatively, chemical chaperones which stabilise PrP^C^ structure (Cortez and Sim, 2013) or compounds which prevent interaction of PrP^C^ with PrP^Sc^ could be used to prevent further protein misfolding (Caughey and Race, 1992; Caughey and Raymond, 1993; Priola et al., 2000). A novel approach targeting the UPR has reported clinical improvements in prion-infected mice (Moreno et al., 2013). This study used GSK2606414, a potent PERK inhibitor, to reduce the chronic phosphorylation of eIF2α and reverse the depression of protein translation that contributes to prion toxicity (Moreno et al., 2012). Interestingly, clinical improvements were evident despite little effect on the level of PrP^Sc^. Despite these encouraging findings, prion pathogenesis is likely to be multi-factorial, with many elements contributing to toxicity (Aguzzi and Falsig, 2012). Hence, treatments aimed at the primary toxic insult (i.e., prion conversion and PrP^Sc^ accumulation) should be effective in treating all aspects of toxicity. Reducing PrP^Sc^ load by stimulating cellular degradation pathways (Fig. 2) could therefore, represent an effective therapeutic strategy.

Consistent with this hypothesis, several studies have reported that upregulating PrP^Sc^ degradation can lead to significant clinical benefit. A series of reports have shown that autophagy induction leads to both PrP^Sc^ clearance in cell models and more importantly, increased lifespan in prion-infected mice (reviewed in Yao et al., 2013). Treatment with rapamycin was shown to activate autophagy in vitro and delay disease onset in mice with prion disease (Cortes et al., 2012; Heiseke et al., 2009). Similar effects were reported using compounds which activate autophagy through mTORC-independent pathways. In prion-infected mice, trehalose was shown to delay the appearance of PrP^Sc^ in the spleen (Aguib et al., 2009) and lithium was found to increase lifespan (Heiseke et al., 2009). The relatively modest improvements reported may reflect the difficulty in achieving the necessary drug concentrations in vivo due to poor blood brain barrier penetration, or simply because the effective concentrations of these drugs are particularly high. It should also be noted that the correlation between PrP^Sc^ clearance and the stimulation of autophagy was based primarily on preliminary in vitro experiments. It is therefore possible, that the above compounds achieved beneficial effects through modulation of non-autophagic pathways (Aghdam and Barger, 2007; Maiese et al., 2013).

Several drugs originally used to target unrelated pathways have also been found to stimulate autophagy and reduce prion disease severity in mice. Treatment of prion-infected mice at 20 days post-inoculation with FK506, a well-known immunosuppressant drug, resulted in an upregulation of autophagic markers, a reduction in PrP^Sc^ levels and an extension in lifespan (Nakagaki et al., 2013). Resveratrol, a phytoalexin enriched in grapes was shown to activate Sirt1, induce autophagy and protect against prion-mediated toxicity, both in cell culture (Jeong et al., 2012; Seo et al., 2012) and in an in vivo C. elegans model (Bizat et al., 2010). The plant extract sulforaphane was originally found to act through the Nrf2 pathway to protect against oxidative stress (Chapple et al., 2012). Recent reports have demonstrated that sulforaphane treatment prevents against prion neurotoxicity in cell culture models (Lee et al., 2014) and induces autophagy in vivo (Liu et al., 2014). Interestingly, this drug was also shown to activate the UPS (Gan et al., 2010; Kwak et al., 2007; Liu et al., 2014), making it an attractive anti-prion agent.

Increased lysosomal breakdown of PrP^Sc^ through autophagy-independent pathways could represent an alternative therapeutic avenue. Branched polyamines are a class of compounds with well-established anti-prion activity in cell culture models (Supattapone et al., 1999; Supattapone et al., 2001). On administration to prion-infected mice, they were shown to slow the accumulation of splenic PrP^Sc^ following intraperitoneal prion inoculation (Solassol et al., 2004). These compounds bind PrP directly and are thought to facilitate lysosomal degradation of PrP^Sc^, possibly by breaking up aggregates in the acidic lysosomal environment (Supattapone et al., 1999). The tyrosine kinase inhibitor STI571, originally developed to treat chronic myeloid leukaemia (Capdeville et al., 2002) has also been shown to have anti-prion activity. This is likely to be through the inhibition of c-Abl which in turn induces lysosomal degradation of PrP^Sc^ through an as yet poorly characterised pathway (Ertmer et al., 2004). Importantly, STI571 treatment at an early phase of peripheral scrapie infection delayed the appearance of PrP^Sc^ in the brain stem and spinal cord and slowed the onset of clinical disease in mice (Yun et al., 2007). Although untested in vivo, tamoxifen is another widely available pharmaceutical that may have therapeutic applications in prion disease. Tamoxifen and its metabolite 4-hydroxytamoxifen were shown to induce the lysosomal degradation of PrP^Sc^ in prion-infected cells, possibly by diverting the trafficking of both PrP and cholesterol to lysosomes (Marzo et al., 2013). A novel approach to upregulate protein clearance is the use of lysosomal modulators (Bahr et al., 2012). Whilst untested in prion disease, these have been shown to increase lysosomal protease expression and activity, and were found to have protective effects in mouse models of AD (Butler et al., 2011; Viswanathan et al., 2012). Their development has come from the surprising observation that mild lysosomal protease inhibition induces the expression of not only the specific enzyme target, but also other unrelated proteases (Bahr et al., 2012). This leads to a global increase in lysosomal enzyme activity and alleviates protein accumulation and toxicity in disease models (Viswanathan et al., 2012).

Although potentially an attractive target for anti-prion therapies, the UPS has so far proved intractable as a drug target. To date, only one bone fide activator has become available. This drug, IU1, is a specific inhibitor of the 19S proteasome-associated ubiquitin chain trimming enzyme, Usp14. Inhibition of this enzyme blocks substrate deubiquitination and enhances its degradation. Increased degradation of disease associated forms of tau, TDP-43 and ataxin-3 in cell culture models have been reported (Lee et al., 2010). Although untested in vivo, IU1 highlights the potential for therapies targeting UPS activity. Manipulations aimed at increasing the catalytic activity of the 20S proteasome through genetic upregulation of various subunits or small molecule enhancers have been reported but their significance in vivo may be limited (Dantuma and Bott, 2014; McKinnon and Tabrizi, 2014). One exception is sulforaphane, which has been shown to stimulate all three proteasome peptidase activities in brain extracts from drug treated mice (Liu et al., 2014). In addition, the levels of ubiquitinated proteins and a UPS reporter construct were reduced in the brains of these animals. In vitro, sulforaphane increased mtHtt degradation and protected cells against mtHtt toxicity; an effect which was abrogated by proteasome inhibition (Liu et al., 2014). To date, the efficacy of sulforaphane against prion disease remains untested. Its ability to stimulate both the UPS and autophagy (Liu et al., 2014) make it an attractive anti-prion agent.

An alternative approach is to augment UPS activity by stimulating the action or expression of chaperone proteins with small molecule compounds (Dantuma and Bott, 2014). Chaperones counteract aggregation, unfold potential UPS substrates and present them in a form readily degraded by the proteasome. Protective effects of such molecules have been reported in animal models of spinal-bulbar muscular atrophy (SBMA) and amyotrophic lateral sclerosis (ALS) (Kalmar et al., 2012; Malik et al., 2013). Once again, these compounds are yet to be tested in prion disease models.

## 7 Perspectives

Although no effective treatment exists for prion diseases, many pathways have been identified that could be targeted for therapeutic intervention. Prion degradation pathways can be included in this group. There is good experimental evidence from in vivo and in vitro studies that pharmacological induction of lysosomal activity clears PrP^Sc^ from neuronal cells and has a protective effect against prion disease pathogenesis. In particular, the benefits of compounds that induce autophagy are well documented. It seems likely that reagents stimulating the UPS could play a similar role. However, small molecules capable of doing this in vivo have yet to be fully characterised and their efficacy in prion disease models remains largely untested. Although still at the experimental level, approaches targeting PrP^Sc^ degradation, in combination with other promising methods, may provide effective therapeutic and/or prophylactic treatments against prion diseases.
